# Research on climate change and mental health in immigrants is urgently needed: A systematic scoping review

**DOI:** 10.1016/j.joclim.2025.100605

**Published:** 2025-11-25

**Authors:** Sasha Houlden, Angela Dawson, Fiona Charlson, Andrew Hayen, Ying Zhang

**Affiliations:** a University of Sydney, Australia; b University of Technology Sydney, Australia; c Queensland Centre for Mental Health Research, Australia; d University of Queensland, Australia

**Keywords:** Immigrants, Mental health, Climate change, Disaster response

## Abstract

**Introduction:**

Globally, climate change is an imminent threat to physical and mental health. Climate-related disasters are predicted to increase in frequency, impacting the stability of and access to social systems and public infrastructure, adversely affecting health and well-being. Immigrant populations may be particularly vulnerable to climate change-related mental health impacts. The bidirectional relationship between climate change and migration infers that climate change-related health threats will further influence increasing migration rates. However, there is limited research that explores mental health risk factors and adaptation and mitigation strategies associated with climate change for immigrants.

**Methods:**

A scoping review was conducted based on a systematic searching strategy. The study aimed to identify and synthesise existing evidence to better understand the impact of climate change on the mental health of immigrant populations, and provide recommendations for future research and practice.

**Results:**

Findings are limited by the quality and depth of existing literature on the topic, as only eight original publications were identified for inclusion in the scoping review, all of which were either qualitative by design or perspective pieces. There is a paucity of evidence on the mental health outcomes of immigrant populations, limiting the recommendations for improving climate-related disaster preparedness and response efforts for immigrants.

**Conclusion:**

Future research and the development of data collection systems that capture health indicators of immigrants are needed to assess immigrant vulnerability to climate-related mental health outcomes.

## 1 Introduction

Climate change is associated with an increasing frequency of natural disasters, desertification, coastal degradation, increased temperatures, pollution and altered food production methods, leading to changes in food availability and disease distribution, which inflict negative economic and health impacts on the global population [1]. Climate change has both physiological and psychological impacts [2,3]. Extreme weather events and natural disasters disrupt the stability of and access to transportation, medical, educational, and social systems and infrastructure. This can lead to heightened levels of anxiety, depression, post-traumatic stress disorder (PTSD), substance abuse, and domestic violence, consequently impacting psychological well-being [4]. ^.^Specific groups may be at a heightened risk of adverse psychological health effects from climate-related adverse weather events, including the elderly, children, those of low socioeconomic status, Indigenous persons, communities from culturally and linguistically diverse (CALD) backgrounds and immigrants [5].

**Fig. 1:**
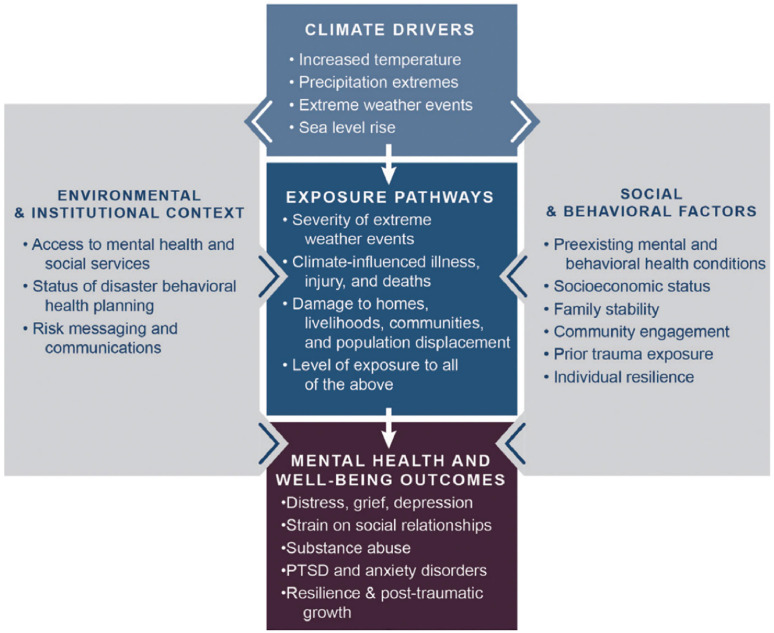
Climate change and mental health and wellness model [6].

The impact of climate change on mental health is complex in terms of underlying sociocultural, economic and ecological determinants. Balbus et al. developed the Climate Change and Mental Health and Wellness Model (CCMHWM) as a framework (Fig 1) to identify key exposure pathways to health threats to mental health and wellbeing due to climate drivers [6]. Climate drivers and factors that influence the climate system, including rising sea levels and temperature, extreme weather events and precipitation extremes, are identified alongside the level of severity of the event such as damage to homes, livelihood and displacement [6]. Social and environmental contexts, such as access to financial resources, health services and communication, are also considered to better understand the impacts on mental health and wellbeing. An integrative review of the literature on the mental health impacts of climate change on vulnerable populations globally has suggested that climate migrants and populations living in areas prone to extreme climate events should receive preventative care before climate-driven events occur to reduce the burden on mental health and wellbeing [7].

Immigrants can face extra challenges of climate change and be more vulnerable to the mental health impacts of climate change. They may be more likely to have already experienced anxiety, stress or mental trauma alongside diminished capacity for adaptation as a result of social and economic disparities [8]. Schutte et al.'s framework presents a bi-directional relationship between health and migration as climate change-related health threats influence migration rates and migration, in turn, impacts health outcomes and wellbeing [9]. Globally, fifty million to 1 billion people are predicted to be displaced by 2050 as a result of the changing climate [10]. Considering the significant risks posed to physiological and psychological health by climate-related factors and migration and their bi-directional relationship, there is a need for further research to explore their connections and contributing risk factors. This will provide important insights to build climate resilience for vulnerable populations [11].

The relationship between climate change and mental health is under-researched, and available studies focus on populations in high-income countries (HIC) and are less focused on immigrants [12]. Reviews of the effects of climate-related disasters on the mental health of populations in low- and middle-income countries (LMIC) suggest that funding and access to mental health services and increased adaptation and resilience planning within the mental healthcare system are required to reduce the negative mental health effects of climate change [13,14]. This study aims to synthesise existing evidence to better understand the impact of climate change on the mental health of immigrant populations, identify gaps and provide recommendations for future research and practice.

## 2 Materials and methods

A systematic scoping literature review was conducted due to the emerging research field that concurrently explores climate change, mental health and immigrant health. In defining the population of interest, immigrants/migrants is used as a general term to include a broader target population, which does not distinguish between climate refugees or climate-forced immigrants from immigrants who have settled in another country not due to climate events. We acknowledge there could be overlap in the experiences and definitions of these populations. Climate change exposures included rising temperatures, extreme weather events and natural disasters including earthquakes. While earthquakes are traditionally classified as geophysical rather than climate-related events, emerging research suggests that climate change may indirectly influence seismic activity [15]. Evidence suggests that processes such as glacial isostatic adjustment, permafrost thaw, and changes in surface water loading resulting from extreme temperatures, precipitation, or drought can alter stress distributions in the Earth's crust, potentially triggering seismic events in specific regions.

The methodology for this review adhered to the Preferred Reporting Items for Systematic Reviews and Meta-Analyses (PRISMA) guidelines to ensure transparency in the review process. Publications of all research types, including empirical studies, literature reviews, editorials, dissertations, and book chapters, in English, with no restriction on geolocation, published in the last ten years, which specifically discussed the study population, immigrant populations, climate change and mental health were reviewed.

The search strategy utilised Boolean operators to combine terms within three main categories: climate change-related events, mental health conditions, and migration status. PubMed, APA PsycINFO and APA PsycArticles were searched using a combination of terms which captured articles related to climate change-related events, mental health conditions, and migration status. Search terms used are listed below.

*Climate change-related events and exposure terms*:

Climate change, warming, bushfire*, wildfire*, fire, flood*, drought*, heatwave*, "extreme temperature", hurricane*, cyclone*, thunderstorm, “natur* disaster”

Mental health outcomes and effects terms:

"mental health", "mental wellbeing", mental*, psychological*, "mental distress", depression, "bipolar disorder", "acute stress", schizophrenia, "psychiatric illness", "acculturation stress", anxiety, "post-traumatic stress disorder", "behavioral disturbance", solastalgia

Migration and refugee populations terms:

refugee*, migrant*, immigrant*, "asylum seeker", asylum, “climate migrants”

After the removal of duplicates, an initial relevancy screen of all unique records was conducted to identify those titles/abstracts clearly unrelated to the study objective (i.e., did not have climate-related exposure or discuss climate change, did not have an outcome related to mental health, did not include, or discuss immigrant populations). Two reviewers then thoroughly examined the included full texts to determine whether they met the inclusion criteria. Any conflicts were discussed between the two reviewers until mutual agreements were reached.

Data was then extracted from the relevant articles identified through full-text screening. In this phase, reviewers systematically extracted relevant information from each included article, such as study characteristics, methodology, key findings, and any other relevant data. The findings from included publications are summarised in the results, including the key themes, patterns, and gaps identified in the literature. The reviews were synthesised in narrative form as there was insufficient methodological quality to conduct a quantitative summary of results. To summarise the information obtained from the narrative review in a meaningful format, narrative synthesis also included utilising the Balbus et al. CCMHWM framework in the discussion section for reporting findings in a structured and coherent manner [6].

## 3 Results

**Fig. 2:**
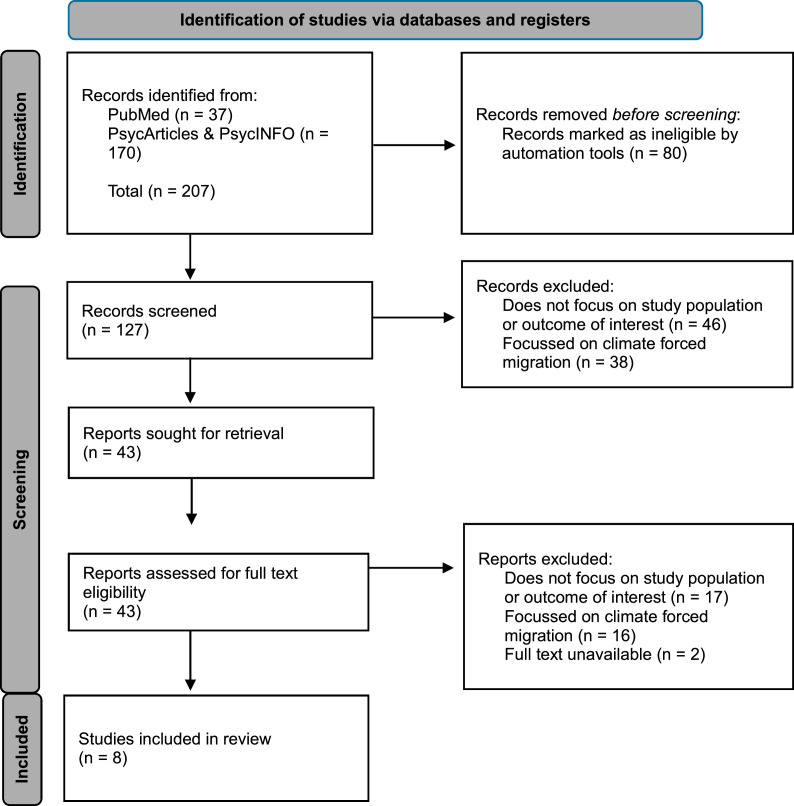
PRISMA flow diagram of database and register search.

The initial search yielded a total of 207 articles. After removing duplicates and applying inclusion and exclusion criteria, eight publications were identified for inclusion in the review. The identification, screening and inclusion process is presented in the flow chart in Fig. 2.

The eight publications included two book chapters, two original studies, one systematic review, an editorial piece, and two dissertations. A summary of the characteristics of the included publications is presented in Table 1. Half were conducted among immigrants in the United States, one in Italy and Nepal, and two studies were global. The study designs were mostly qualitative, with a lack of quantitative analysis. Mental health issues (e.g. PTSD and trauma) after earthquakes were the most studied outcome. The main themes discussed in the articles were increased risks of climate change-related adverse mental health outcomes for immigrant populations, improving disaster literacy and preparedness, cultural barriers affecting mental health care access after a disaster and the influence of community dynamics [[16], [17], [18], [19], [20], [21], [22], [23]]. Diverse immigrant settings and their adaptation to climate disasters were largely missing in the literature, indicating a strong need for more research to understand the scale of the problem and solutions to protect mental health among immigrants from the changing climate.

**Table 1:** Results summary of included publications.

Title	Year	Country	Publication types	Methods	Findings	Implications
The intersection of trauma and disaster behavioral health: Evidence-Based Treatment for Mental Health Care Post-Disaster [16]	2021	USA	Book chapter	Perspectives	• Data literacy is outlined as an important factor disaster preparedness for immigrants who may not speak the dominant language fluently where they live. • Considering cultural and literacy factors and the inclusion of populations from CALD backgrounds in disaster preparedness efforts improves disaster response outcomes.	Disaster literacy and disaster preparedness for CALD communities
Integrating technology into modern therapies A clinician's guide to developments and interventions: Videoconferencing in Psychotherapy [17]	2019	USA	Book chapter	Perspectives	• Psychotherapy videoconferencing is recommended as a crisis-oriented mental health care approach to reach immigrant populations.	Mental health care approaches for vulnerable populations
Do remittances alleviate negative impacts of disaster on mental health? A case of the 2015 Nepal earthquake [18]	2019	Nepal	Peer-reviewed original study	Qualitative interviews conducted before and after the earthquake studying the effects of remittances on the mental health of victims of a disaster	• Increased remittances reduced stress and depressive symptoms of family members following the earthquake. • However, this didn't completely alleviate mental health impacts experienced by family members.	The effects of remittances on the mental health of family members whose families have experienced climate related disasters.
Out-group threats and distress as antecedents of common in-group identity among majority and minority group members in the aftermath of a natural disaster [19]	2018	Italy	Peer-reviewed original study	Qualitative interviews of post-earthquake experiences and perceptions among immigrants and Italian nationals to examine the role of out-group threats in fostering one-group perceptions directly and indirectly via post-traumatic stress symptoms in the aftermath of a natural disaster.	• The experience of a climate disaster can exacerbate mental health issues among immigrants, particularly when they perceive threats from the out-group. I • Considering social dynamics and intergroup relations is important for understanding the mental health impacts of climate disasters on immigrant populations.	How out-group threats affect the mental health and perceptions of belonging among immigrant and non-immigrant earthquake survivors
“Forever marked”: A Qualitative Study Exploring Cultural Terminology For PTSD Among Haitian Immigrant Women[20]	2017	USA	Dissertation	Qualitative interviews with Haitian immigrants living in the US to explore experiences of PTSD after the earthquake in Haiti were conducted. PTSD symptoms were assessed using the PTSD Checklist Specific (PCL-S). Acculturation to American and Haitian cultures was measured using the Language, Identity, and Behaviour Acculturation Scale (LIB).	• Haitian immigrants in the US may not feel comfortable expressing their cultural views or perspectives on trauma due to language and cultural barriers.	Culturally specific perceptions of trauma and suffering, spiritual and beliefs, coping strategies, barriers to treatment for immigrants who have experienced natural disasters.
Exploring the relationship between psychological symptoms and ethnic connectedness among the New York Metropolitan Area Haitian community to the 2010 earthquake in Haiti[21]	2016	USA	Dissertation	Cross sectional study with Haitian immigrant residing in the US using questionnaires which measured the psychological impact, including anxiety, depression and vicarious trauma symptoms as a result of the 2010 earthquake in Haiti.	• The importance of understanding the psychological impact of natural disasters on immigrant communities and the need for culturally competent mental health services. • The findings suggest a broader model for understanding the immigrant experience in the United States.	The importance of culturally informed mental health care for immigrants, and the influence of connectedness to immigrant’s home countries on mental health outcomes.
The Impact of Climate Change on Mental Health: A Systematic Descriptive Review[22]	2020	Global	Systematic descriptive review	The review explored the association between classical psychiatric disorders and phenomena related to climate change and extreme weather.	• Refugees, immigrants and ethnic minorities experience severe mental health impacts due to climate change with women in low- and middle-income countries from low-socioeconomic backgrounds also identified as populations at an increased risk.	Climate change impacts the mental health of immigrant populations, although the discussion of the impacts on this population was limited.
Climate change and mental health: time for action and advocacy[23]	2022	Global	Editorial	The impacts of climate change on mental health outcomes are explored within the review, with immigrant status explored as a risk factor for increased risk of adverse mental; health outcomes.	• Immigrant status and cultural distance have been associated with increased risks of psychotic disorders after experiencing climate related disasters. • Advocacy for increased immigrant reception procedures and services that promote optimal health and well-being from mental health professionals.	Impact of climate change on the mental health of immigrant populations and recommendations for increased disaster behavioural health planning.

## 4 Discussion

Immigrants are an understudied population in climate-related mental health research despite being consistently identified as a vulnerable population for adverse mental health outcomes. Our study has yet to identify quantitative analyses or intervention studies focusing on immigrant populations. The small number of identified articles is largely qualitative by design or perspective pieces. Due to this, the review's findings are significantly limited by the quality and depth of existing literature on the topic. There are both direct and indirect mental health impacts of climate change on vulnerable populations, which are contextualised by the cultural and social settings in which they occur [7]. Due to the limited number of articles available on the topic, this discussion will provide an overview of the barriers and challenges faced by immigrant populations in relation to the mental health impacts of the climate crisis, and encapsulate the multidimensionality of the contextual factors structured through the CCMHWM framework by Balbus et al. [6].

### 4.1 Overview of the problem

Overall, there is a lack of empirical evidence to aid the understanding of the mental health impact of climate change on immigrants. Environmental and institutional contexts, including the state of public infrastructure, governance, management and institutions, influence the ability of immigrant populations to adapt and maintain wellbeing with the experience of the changing climate and climate-related disasters [6]. These factors affect both individual and community vulnerability and adaptive capacity, sensitivity and exposure to climate change. Disaster behavioural health planning is essential for disaster preparedness and response, as experiencing a climate-related disaster can lead to the deterioration of social support [24]. Access to timely psychological first-aid services and social support systems is crucial as individuals who are experiencing psychological distress may be difficult to detect and therefore, may not receive appropriate care [2].

Disaster preparedness efforts which factor cultural and literacy differences and include minority groups including immigrant populations are suggested to improve disaster response outcomes as these populations are increasingly vulnerable during climate related disasters [25], [26]. Language barriers and diverse cultural perceptions of mental health are also barriers for immigrants accessing mental health care as well as mental health stigma, economic constraints or fear of negative repercussions in relation to their migration status [27]. Immigrant women who are the primary caregivers to children may experience further barriers to care due to reduced capacity for individual health prioritisation [28].

In addition to these challenges, many immigrant communities require culturally informed mental health care, which recognises the diverse experiences and expression of psychological symptoms outside of the Western- models of mental health care. Different terminology may be used to describe experiences of trauma and depression, which may pose barriers to treatment for culturally diverse immigrant communities facing the impacts of climate-related disasters [20]. Alternative solutions for increasing access to mental health care for vulnerable populations are needed.

### 4.2 Key factors and components of the problem

Social, demographic and behavioural factors also affect individual and community vulnerability and adaptive capacity, sensitivity and exposure to climate change, including socioeconomic status, pre-existing mental health conditions, and family and community support systems [11,27,28]. Vulnerable populations such as immigrant communities may already experience socioeconomic disadvantage, increased life stressors and a reduced sense of autonomy, which is then compounded by the experience of a climate disaster [24]. For example, immigrant populations and recent immigrants, in particular, are likely to have reduced financial freedom and also may not receive the same financial aid awarded to citizens in the event of a disaster [29]. Migration interferes with one’s attachment to place, sense of security, community and identity, which impacts mental health and wellbeing [[30], [31], [32]]. Social and community dynamics and family support influence the mental health outcomes of communities following the experience of a climate-related event. With greater levels of family support, social and community level cohesion are suggested to be a predictive factor for more positive mental health outcomes [33,34]. Although the experience of a disaster as a community can unite the community as one group, conversely, it may increase intergroup conflict, including the exclusion of minority groups such as immigrants due to decreased trust between community members and social and community level cohesion [35,36].

Individuals with existing mental health issues are increasingly at risk of poor mental health outcomes due to climate related events [37]. Climate related disasters often disrupt access to mental health services and resources, which individuals with pre-existing mental and behavioural health conditions are reliant on, decreasing the effectiveness of established coping strategies [38]. The experience of previous traumatic events and climate related disasters also increases the probability of developing adverse mental health outcomes after exposure to a climate-related disaster, with those exposed to multiple disasters increasingly at risk [39]. Immigrant populations are at an increased risk of developing mental health conditions due to the associated emotional, physical and social effects of relocation and may have prior trauma exposure as a result of other events, including crime, war, violence or stressors such as socioeconomic disadvantage or social stigma [38,40,41].

### 4.3 Recommendations and future directions

There is a growing body of evidence that highlights the increased mental health risks associated with climate change. The most studied climate change exposures included multiple natural disasters such as floods, drought, earthquakes, typhoons, and tsunamis [14,[42], [43], [44], [45], [46], [47], [48], [49], [50], [51], [52], [53], [54], [55], [56], [57], [58], [59], [60], [61], [62], [63], [64], [65], [66], [67], [68], [69], [70], [71], [72], [73], [74], [75]]. Vulnerable populations include forcibly displaced migrants, rural populations, children and adolescents, women, and people with a low socioeconomic status [22,36,42,46,53,54,58,68,75].

However, research on immigrants’ health in the context of climate change is very limited, even though immigrant and CALD communities have been reported as populations increasingly at risk of climate disasters [22,25,45,56,63]. More evidence is available about increased population displacement due to changing climates and environments, but this information is not focused on settled immigrants and how ethnicity could contribute to their vulnerability. In addition, the mental health outcomes of immigrant populations have been sparsely addressed, if at all, with limited recommendations for improving climate-related disaster preparedness and response efforts.

Successful strategies for building climate resilience in marginalised communities must be culturally relevant [76]. Resilience to climate-related disasters is informed by individual resilience, which has been suggested to be both a process and a trait most influenced by adaptability and sense of place, followed by financial capacity, family and community support, communications and climate change knowledge and trust in communication sources [77]. Individual resilience both influences and is influenced by family resilience and community engagement [78]. Increasing individual resilience is a crucial preventative approach for reducing the mental health impacts of climate change, especially for vulnerable communities [76]. Available cultural and language support for immigrants attempting to access mental health care is vital at every stage of the process of seeking and receiving mental health care to improve access to mental health care for populations from CALD backgrounds [79]. Unresolved language barriers at all stages of the care pathway, but especially in the early stages, impact the diagnosis and treatment of mental health conditions [79].

More evidence, including mental health impact assessments related to climate change, is needed to support the development of effective interventions to protect immigrants’ health. In particular, research co-designed with community members is necessary to meet the real needs of marginalised populations in responding to climate change. Engaging marginalised community members, such as immigrants, to share their lived experiences and their traditional knowledge promotes individual empowerment and fosters individual resilience to the impacts of future climate events [80]. Community-led post-disaster mental health response programs may be beneficial for ensuring minority groups such as immigrants receive appropriate mental healthcare following a disaster. For example, the Skills for Life Adjustment and Resilience (SOLAR) has been developed to be delivered by non-mental health professionals after a short period of training and can be delivered to individuals or groups to increase access to post-disaster care for communities [81].

Capacity building for mental health professionals and carers should be strengthened by taking a culturally sensitive approach. Despite the growing research on the mental health impacts of climate change, mental health clinicians and practitioners often lack adequate training to effectively support communities experiencing mental health issues as a result of climate change [82]. The Lancet Countdown has outlined the “Inclusion of health and climate change within medical and public health curricula” as an indicator that should be monitored annually, although measuring progress in this area has been difficult [83,84]. Currently, a limited number of climate health professionals are available for guidance and educational training programs for climate-related mental health, with a lack of funding for climate-related health educational programs [82]. The mental health impacts of climate change are yet to be classified directly by the Diagnostic and Statistical Manual of Mental Disorders (DSM-5) and International Classification of Diseases (ICD-10), which may also pose challenges in addressing these growing mental health challenges across various population settings [44].

## 5 Conclusions

The impacts of climate change on the mental health of immigrants remain underexplored, even though there is increasing evidence on the overall mental health impacts of climate change. The bidirectional relationship between climate change and mental health in immigration further denotes the need for culturally sensitive research to inform strategies to improve responses to climate-induced mental health challenges among immigrant populations. Further research is required to determine the scale and the scope of the problem across different settings and develop an integrative framework to guide research, policy and practical interventions. Improving climate-related disaster preparedness will differ in different regions, and therefore, multidisciplinary, international collaboration is required. Future research and the development of data collection systems that capture health indicators of immigrants are needed to reduce immigrant vulnerability to the mental health impacts of climate change and evaluate the implementation of mental health interventions and policies.

## Funding source

The study has not received any funding.

## CRediT authorship contribution statement

**Sasha Houlden:** Writing – review & editing, Writing – original draft, Resources, Methodology, Formal analysis, Data curation. **Angela Dawson:** Writing – review & editing, Resources, Methodology. **Fiona Charlson:** Writing – review & editing, Resources, Methodology. **Andrew Hayen:** Writing – review & editing, Resources, Methodology. **Ying Zhang:** Writing – review & editing, Supervision, Resources, Methodology, Investigation, Data curation, Conceptualization.

## Declaration of competing interest

The authors declare that they have no known competing financial interests or personal relationships that could have appeared to influence the work reported in this paper.

The author is an Editorial Board Member/Editor-in-Chief/Associate Editor/Guest Editor for this journal and was not involved in the editorial review or the decision to publish this article.
